# Perceived Causal Problem Networks: Reliability, Central Problems, and Clinical Utility for Depression

**DOI:** 10.1177/10731911211039281

**Published:** 2021-09-01

**Authors:** Lars Klintwall, Martin Bellander, Matti Cervin

**Affiliations:** 1 Stockholm University. Stockholm, Sweden; 2 Karolinska Institute, Stockholm, Sweden; 3 Lund University, Lund, Sweden

**Keywords:** depression, case conceptualization, cognitive behavioral therapy, CBT, network analysis, comorbidity, nosology

## Abstract

Personalized case conceptualization is often regarded as a prerequisite for treatment success in psychotherapy for patients with comorbidity. This article presents Perceived Causal Networks, a novel method in which patients rate perceived causal relations among behavioral and emotional problems. First, 231 respondents screening positive for depression completed an online Perceived Causal Networks questionnaire. Median completion time (including repeat items to assess immediate test–retest reliability) was 22.7 minutes, and centrality measures showed excellent immediate test–retest reliability. Networks were highly idiosyncratic, but worrying and ruminating were the most central items for a third of respondents. Second, 50 psychotherapists rated the clinical utility of Perceived Causal Networks visualizations. Ninety-six percent rated the networks as clinically useful, and the information in the individual visualizations was judged to contain 47% of the information typically collected during a psychotherapy assessment phase. Future studies should individualize networks further and evaluate the validity of perceived causal relations.

Depression is a psychiatric diagnosis primarily defined as a persistent low mood and/or loss of pleasure. Individual treatment response to both pharmacological and psychological evidence-based interventions vary, likely due to patient comorbidity and the heterogeneity of the diagnosis (Tunvirachaisakul et al., 2018).

Most psychotherapy research has been focused on evaluating disorder-specific treatment manuals, and there is an increasing awareness of the need to acknowledge the diversity of depression and to individualize treatments based on individual factors (Maj et al., 2020). Psychotherapy can be individualized through client-specific case conceptualizations where central maintaining and pathological processes unique to the individual are identified. Case conceptualizations can be highly formalized (e.g. Haynes et al., 2020), enabling statistical analysis, which has been demonstrated using, for instance, the commonly used case conceptualization method of functional analysis (Burger et al., 2020).

Although there is little evidence that conceptualizations actually improve treatment effects, it has been suggested that individualization may be most important when patients have comorbid disorders, which has not typically been the case in studies investigating manualized treatments (Kamphuis et al., 2020). It is also possible that psychotherapist-generated case conceptualizations are less valid than assumed. Indeed, studies investigating interrater reliability for case conceptualizations warrant serious caution (Bucci et al., 2016). Research has also shown that selection of treatment targets is heavily influenced by theoretical allegiance (Kim & Ahn, 2002). Furthermore, case conceptualizations are time-consuming and can divert focus away from testing and evaluating interventions.

One way to improve the clinical use of case conceptualizations is suggested by the network approach to psychopathology (Borsboom, 2017). In the network understanding of mental disorders, symptoms are not seen as indicators of an underlying latent disorder. Rather, symptoms are considered to be causally and reciprocally connected in a system that might stabilize into a pathological state, such as depression. Even though two patients present with the exact same symptoms, the causal relations among those symptoms may differ considerably. Of special interest in personalized symptom networks is the identification of self-reinforcing feedback loops among symptoms (e.g., between anxiety and social withdrawal). Such feedback loops are seen as maintaining a pathological network state (i.e., depression) after it has been activated by, for instance, a negative life event (Borsboom, 2017; Wittenborn et al., 2016).

The network approach to psychopathology has been formalized in the estimation of psychological networks, and a vast majority of studies has been based on cross-sectionally collected item-level data (e.g., the specific items of a depression questionnaire). In this type of network, each item represents a node in the network, and the unique associations among nodes (referred to as edges) are commonly estimated by computing partial correlations between the items (i.e., the associations that remain after all other linear associations in the full set of items have been accounted for). When the partial correlation structure has been estimated, the partial correlations for each specific item can be summed into a score that is referred to as centrality. Items with high centrality have many and strong unique associations with other items in the network. Centrality has been highlighted as important as it may indicate which symptoms that are influential in the development or maintenance of a disorder. Nevertheless, between-subject (i.e., nomothetic) networks based on cross-sectionally collected data are might fail to uncover causal and reciprocal mechanisms on the individual level. For the latter, idiosyncratic networks (i.e., that reflect processes unique to an individual) need to be estimated.

Idiosyncratic symptom networks can be created by different methods, and the most common is to collect dense time series data using ecological momentary assessments (EMAs). This is done by selecting relevant symptoms (or other problematic behaviors or emotions) which the respondent then rates for up to 10 times daily over several weeks (Robinaugh et al., 2020). An autoregressive model on the individual patient data reveals temporal regularities in symptom fluctuations. In its simplest form, using lag-1 correlations, variation in one symptom is associated with variation in all other symptoms at the preceding timepoint, thus, identifying how symptoms precede each other. These correlations can be visualized as networks, with symptoms as nodes and correlations as directed edges (i.e. causal arrows).

As described above, one way to analyze nodes in networks is to assign each node a centrality score, indicating how much the symptom influences other symptoms in the network. A common way to do this is to estimate out-degree centrality, in which the centrality of the node is the sum of all outgoing edges from that node, indicating how much it influences the rest of the network. Although centrality is often used as an indicator on where to intervene on a network, centrality has been criticized both from a conceptual standpoint (e.g., how a sum of correlations should be understood; Bringmann et al., 2019), and from a clinical utility standpoint (e.g., due to nonlinear effects, central nodes need not be optimal treatment targets; Henry et al., 2020).

Few studies investigating whether centrality can help improve treatment outcome exist, but in a recent study, Fisher et al. (2019) collected individual patient data (on average 111 data points per patient during a 1-month pretreatment assessment phase) and used this to adapt the order in which modules in the Unified Protocol treatment manual were delivered. This increased the treatment effect by 35% compared with what could be expected from previous studies using the same treatment manual with similar samples. Although this was an uncontrolled study (i.e., the obtained treatment effect could be due to the sample, quality of the treatment delivery, and so on), it raises the possibility that an idiosyncratic symptom network can be used to successfully individualize treatment.

However, there are some limitations to EMA methods. First, symptoms likely influence each other on timescales ranging from seconds (e.g., worrying causing anxiety) to days (e.g., physical inactivity causing lack of energy). This means that measures must be rated with very high density and all possible time lags need to be analyzed (Robinaugh et al., 2020). Higher time-density results in that fewer symptoms can be rated at each assessment point, potentially limiting the clinical usefulness of the method. Items must be chosen carefully in collaboration with the client (von Klipstein et al., 2020), further adding to the time needed. Finally, a potential limit in the use of EMA is the difficulty of detecting *avoidance* of an aversive emotion or behavior as the cause of other problems. For instance, a client might isolate socially to avoid panic attacks, and this successfully results in few experiences of panic. In a clinically relevant sense, panic attacks is thus the cause to the client isolating, although panic attacks rarely actually precede isolation, a situation which would be hard to detect using EMA.

An alternative to EMA for creating client-specific symptom networks is the perceived causal relations (PCR) scaling methodology, first described in Frewen et al. (2012). In PCR, the respondent selects relevant symptoms from a list and then rates the extent to which every selected symptom causes every other selected symptom. Using a sample of undergraduate students (*N* = 225) and a list of symptoms of PTSD, depression, and anxiety disorders, Frewen and colleagues found that anxiety and traumatic memories tended to be causes, on a group level, of depressive symptoms, rather than the other way around (Frewen et al., 2012). In a second study, the number of feedback loops in individual networks was found to predict symptom frequencies, as expected from network theory (Frewen et al., 2013). The PCR methodology overcomes some of the limitations of other network analysis methods. It does not suffer from the nomothetic nature of between-subject networks. Furthermore, it is more time-efficient than EMA, can include more symptom information, and uncover causal relations not easily detected using EMA (e.g. from the issues with time-scales or avoidance, described above). Accordingly, structured self-reported PCR case conceptualizations hold promise as a useful first step in creating idiosyncratic case conceptualizations, which could be further elaborated in collaboration between therapist and client. This method has been described as promising and underutilized (Robinaugh et al., 2020). However, neither the clinical utility of idiosyncratic PCR networks using behavioral/emotional problems (as opposed to list of symptoms), nor the reliability of these networks have been investigated.

The purpose of the present study is to investigate whether a clinically adapted version of the PCR method, Perceived Causal Networks (PECAN; including visualizations of individual results), is a reliable and useful method to create idiosyncratic networks of emotional/behavioral problems for adults with depression. Specifically, we aim to investigate the immediate test–retest reliability and average time needed to complete PECAN, explore to which degree the PECAN networks vary across participants and investigate how psychotherapists rate the clinical utility of PECAN networks, for what purpose in an assessment phase the method would be useful, and how the method could be improved.

## Method

### Design

We used a two-step design to evaluate the PECAN methodology. In Study 1, respondents who screened positive for depression completed an online version of PECAN twice to assess test–retest reliability, time to complete the questionnaire, and similarities/differences across participants. Although not necessarily the best metric to select treatment targets, node centrality was chosen as the main metric of interest to assess reliability. In Study 2, the clinical utility of PECAN was explored by asking psychotherapists to rate randomly selected networks from Study 1 for usefulness and report what information was missing from PECAN. Although this does not assess the validity of the idiosyncratic networks, it was deemed a necessary first step in making the method clinically useful. The study was approved by the Swedish Ethical Review Authority (ID 2020-06113). We report how we determined our sample size, all data exclusions, all manipulations, and all measures in the study. Since the purpose was exploratory rather than hypothesis-testing, no preregistration was conducted.

## Study 1

### Method: Study 1

#### Participants

Adverts were posted in 56 Swedish Facebook groups related to mental health. Data were collected for 1 month, without predetermined required sample size. In total, 992 individuals clicked the link to the online questionnaire. Of these, 39% terminated participation during the study information/training stage, 25% terminated participation during ratings of perceived causal relations and background items, and 36% (355 respondents) completed the full questionnaire. Of these, 116 were excluded due to a PHQ-9 score < 10 and another eight due to stereotypic responding (defined as a sudden and persistent switch during retest to at least three consecutive causal relations items being rated as 0). The final sample consisted of 231 respondents (90% female, 9% male, and 1% other genders) with a mean age of 39.4 years (*SD* = 12.9), of which 54% had a university degree and 54% had experience of cognitive behavioral therapy (CBT). The mean PHQ-9 score was 17.0 (*SD* = 4.6).

#### The Online PECAN Questionnaire

##### Informed consent

Respondents were informed about the purpose of the study and were guaranteed anonymity. Each respondent actively consented to participate.

##### Selecting relevant behavioral/emotional problems

A list of 26 items (behavioral/emotional problems) were presented (see Table 2) and respondents were asked to select items that they had experienced during the past week. The present pool of behavioral/emotional problems was selected based on piloting of different versions of the questionnaire and settling on a list that yielded both acceptable reliability and high therapist ratings of utility (the list could and should be adapted to diverse clinical populations in which the PECAN might be used in the future). Limiting the list to 26 items was done to decrease the risk of overwhelming respondents. Respondents were asked to select between seven and 15 items (pilot data showed that fewer selected items yielded networks that were not deemed clinically useful; allowing more selected items resulted in unacceptably long completion times).

##### Rating severity

Each selected item was rated for “severity” on a 0-to-100 scale. Severity was described as “How disturbing is this problem for you, in itself?” corresponding to the “estimated relative importance of behavior problems” as described by Haynes et al. (2020).

##### Training trials

To ensure that the respondent understood the causal relation questions as intended, three multiple choice training questions were provided. These were examples of situations when an event with a clear direct cause occurs (as opposed to, e.g., teleological causes). The respondent was allowed to continue with the questionnaire only when she or he had answered the training questions correctly.

##### Rating perceived causal relations

The respondent was then asked to report to what degree each selected item was caused by every other selected item (corresponding to the “estimated magnitude of effect” in Haynes et al., 2020). For each item, the respondent was presented with a list of every other selected item and asked to select no more than three of these items as causes. The respondent could also select “none” and continue. Only positive relations were assessed (one behavior/emotion *increasing* another behavior/emotion), not negative (one behavior/emotion *decreasing* another behavior/emotion). When selecting the causes of emotions (Items 18 through 26 in Table 2), other emotions were not included as optional causes because pilot data had showed that emotion-to-emotion causality resulted in networks with less clinical utility as rated by psychotherapists trained primarily in behavior therapy. This limitation could be excluded in future versions of the questionnaire, given that other therapeutic traditions might consider emotion-to-emotion causal relations to be more clinically relevant. If one or more items were selected as causes for an item (e.g. the respondent selected “worrying” and “substance use” as causes for “sleep problems”), the respondent was asked to distribute percentages across these items as well as for an option termed *other causes* / don’t know, indicating the perceived causal strength of each relation (e.g. the respondent might allocate the causes of “sleep problems” as 30% caused by “worrying”, 60% by “substance use”, and the remaining 10% by “other causes / don’t know”). The distribution of a sum-total of 100% across causes was found during piloting to be a way to deal with the issue of some respondents otherwise scoring almost all items as 100% caused by every other item, resulting in low clinical utility. Again, not more than three causes could be selected for each item. To facilitate completion, items were always presented in the same order (the order shown in Table 2). Items were described with slightly different wording when presented as being caused versus causing a problem (as shown in Appendix A; available online). Of note, avoidance was included in the description of some items as a cause (as seen in Table 2). For example, when the “sad/alone” item was presented as a cause, it was phrased “I felt, *or wanted to avoid feeling*, sad or alone.”

##### Immediate test–retest

Severity ratings and ratings of perceived causal problem relations were repeated as part of the same response session, but respondents were not asked to reselect items.

##### Depressive severity and background information

Respondents completed the PHQ-9 and reported on gender, age, education level, and experience of CBT. Finally, each respondent was provided with a randomized code which could be used to retrieve the individualized problem network (which 52% of respondents did).

#### PHQ-9

The Patient Health Questionnaire (PHQ-9) covers the *DSM-5* criteria for major depression. Each item is scored on a 0-to-3 scale, yielding a total score of 0 to 27. A cutoff point of 10 has been shown to yield a specificity of 0.89 and a sensitivity of 0.85 for identifying major depression (Manea et al., 2012).

#### Data Analysis

##### Completion time

We report both completion time and completion time divided by number of selected items. Because of a skewed distribution (some respondents had very long completion times), times for completion are reported using medians and interquartile ranges.

##### Item weighted outdegree centrality

**Figure 1.**
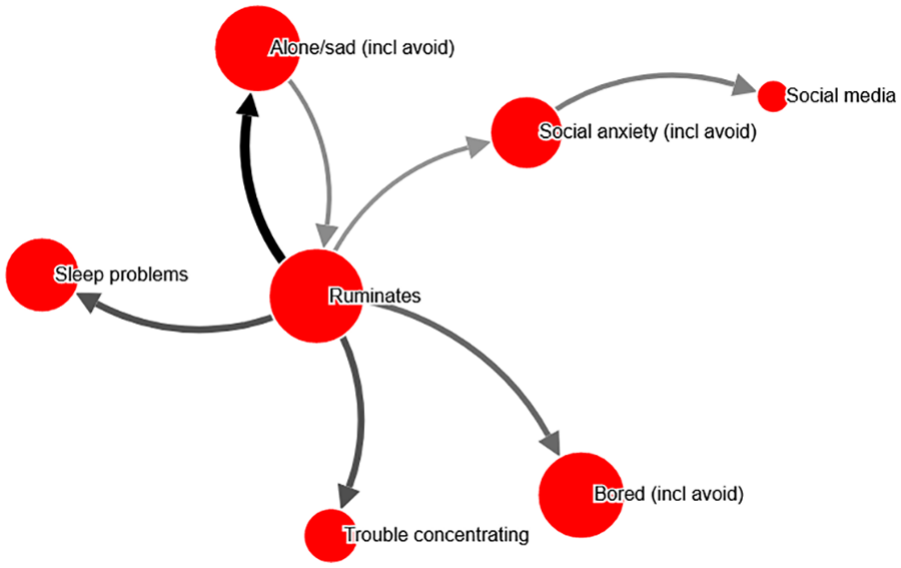
Perceived causal problem network, example from dataset to exemplify calculations, chosen due to simplicity (shown in Table 1). *Note*. This network (ID 995759) is for a female respondent with a PHQ-score of 12, aged 40 to 49 years, with a test–retest (r) of .97. For simplicity, perceived causal relations weaker than 40% have been omitted in both visualization and the example computations in Table 1. An interactive version can be found at http://bit.ly/PECANfig1

For each respondent, selected items were given centrality scores. Out-degree centrality (i.e., the sum of all outgoing relations) is a standard centrality measure in the network literature (Robinaugh et al., 2020). However, since item severity varied considerably, out-degree relations for a specific item were weighted by the severity of the items to which it was connected. In other words, the centrality of each item reflected the sum of that item’s severity, the severity of all items that it had an outgoing causal edge to, and the percent ratings of those causal relations. Although this is a novel variant of the more standard out-degree centrality, including the importance or severity of each problem in the calculation of item centrality, this was done to improve clinical utility of the measure. Furthermore, we report proportional item centrality, that is, the centrality for a specific item divided by the sum of the centrality of all items in the network. An example of how proportional centrality was calculated is presented in Figure 1 and Table 1.

**Table 1.** Example computations for network shown in Figure 1. Although relations weaker than 40% are emitted for these calculations, all relations were included in actual computations in the study.

Selected items	Severity (node size)	Severity-weighted outdegree (arrow thickness indicate percent ratings)	Node centrality (node severity + weighted outdegree)	Proportional node centrality (% of total network centrality)
Sleep problems	69	Not causing other items	69	9.2
Trouble concentrating	50	Not causing other items	50	6.6
Social media use	29	Not causing other items	29	3.8
Ruminates	89	Causing alone/sad: 100% of 81 severity = 81 Causing insomnia: 67% of 69 = 46 Causing social anxiety: 41% of 67 = 27 Causing anhedonia: 58% of 81 = 47 Causing unfocused: 68% of 50 = 34 Total outdegree = 235	89 + 235 = 324	43.0
Social anxiety	67	Causing social media use: 49% of 29 = 14	67 + 14 = 81	10.7
Alone/sad	81	Causing rumination: 44% of 89 = 39	81 + 39 = 120	15.9
Bored	81	Not causing other items	81	10.7
TOTAL	**466**		**754**	100%

##### Immediate test–retest

For each respondent, two measures of test–retest reliability were calculated: for problem centralities and for all relations. Spearman correlations were used to estimate test–retest correlations.

### Results: Study 1

#### What Is the Immediate Test–Retest Reliability of PECAN?

For item weighted outdegree centrality, the average immediate test–retest correlation was .81 (*SD* = 0.14). For perceived causal relations between items, the average immediate test–retest correlation was 0.53 (*SD* = 0.20). For average reliability of specific items, see Table 2. Note that the reliability for centrality is higher than for individual relations, likely because respondents tended to select the same item as a strong cause, but differing on what other items it caused.

**Table 2.** List of items in current version of PECAN, and results. Note that items 18-26 (marked with *) could not be selected as causes for eachother.

Number	Behavior/emotional problems (items)	Percent selected	Mean severity 0-100 (*SD*)	Mean prop. centrality (*SD*) when selected	Mean percent caused by other problems (*SD*)	Mean test–retest (*r*) for relations in/out	Percent frequency most central
1	Eats less	31	26.8 (26.8)	9 (11)	63.6 (41.7)	.46	0.4
2	No exercise	49	43.0 (27.2)	19 (18)	75.1 (34.4)	.48	3.5
3	Sleep problems	52	55.0 (27.0)	27 (23)	78.9 (34.7)	.55	8.7
4	Daytime resting	39	28.4 (28.4)	10 (12)	77.0 (34.5)	.34	0.4
5	Conflicts	16	60.3 (28.6)	25 (18)	63.5 (42.1)	.62	2.6
6	Hypocondric worries	15	49.7 (31.1)	24 (24)	71.0 (40.5)	.65	1.3
7	Trouble concentrating	54	56.9 (24.9)	28 (18)	87.2 (25.3)	.44	5.2
8	Social media use	53	32.5 (25.0)	15 (15)	75.1 (35.9	.46	0.9
9	Stays at home	20	46.3 (28.8)	16 (14)	82.3 (33.8)	.53	0.9
10	Procrastinates	57	51.5 (26.2)	20 (17)	77.4 (34.7)	.43	3.0
11	Substance use	24	40.9 (33.1)	14 (16)	64.8 (44.0)	.64	1.3
12	Self-harm	5	75.4 (22.7)	21 (14)	100.0 (0.0)	.72	0.4
13	Suicidal thoughts	9	76.0 (28.2)	26 (23)	99.8 (0.4)	.57	1.3
14	Eats more	19	49.5 (31.4)	15 (15)	81.6 (31.9)	.46	1.7
15	Compulsions (incl avoid)	6	63.6 (29.9)	29 (29)	43.5 (43.2)	.63	1.7
16	Ruminates (incl avoid)	57	71.6 (23.0)	40 (22)	76.4 (35.2)	.44	19.5
17	Worries (incl avoid)	62	71.0 (24.2)	41 (23)	73.0 (37.9)	.42	17.7
18*	Flashbacks (incl avoid)	13	81.2 (21.2)	28 (21)	55.3 (45.1)	.50	1.3
19*	Panic (incl avoid)	23	75.2 (22.2)	22 (15)	62.1 (44.7)	.50	1.7
20*	Pain (incl avoid)	53	55.0 (28.6)	19 (17)	47.9 (43.6)	.59	2.6
21*	Social anxiety (incl avoid)	33	61.1 (26.2)	20 (13)	35.9 (42.4)	.44	2.2
22*	Alone/sad (incl avoid)	45	72.4 (21.7)	31 (20)	57.1 (42.5)	.54	7.8
23*	Tired (incl avoid)	60	58.8 (29.7)	24 (18)	60.1 (40.1)	.52	7.8
24*	Stressed (incl avoid)	59	59.1 (27.6)	26 (18)	63.2 (40.6)	.43	3.5
25*	Bored (incl avoid)	50	49.3 (27.9)	17 (15)	64.0 (40.4)	.51	1.7
26*	Angry (incl avoid)	42	50.4 (30.7)	13 (11)	50.6 (42.7)	.50	0.9

#### How Much Time Is Needed to Complete PECAN?

The median time to complete the PECAN was 22.7 minutes (IQL = 18.8). On average, respondents selected 10.4 items (*SD* = 2.8). The median completion time divided by the number of selected items was 2.3 minutes (IQL = 1.6). Note that these numbers would be roughly halved if the retest items were dropped from the questionnaire.

#### To What Degree Do PECAN Results Differ Across Participants?

Each included item was rated as the most central item for at least one respondent (see last column in Table 2). One of the two most frequent items, “Ruminates” and “Worries,” were most central for 37% of the respondents. However, note that items 18-26 could not be selected as causes for eachother, a limitation that somewhat limits possible interpretations of this finding.

Individual edges varied considerably in strength across respondent networks, with the average standard deviation in edge strength (including only edges with a count over 20) being 15.6%.

Data on edge counts, average strengths, and standard deviations can be found in the Supplemental Material (available online).

## Study 2

### Method: Study 2

#### Participants

Adverts were posted in Swedish Facebook groups for psychologists and psychotherapists. Data were collected for 1 month, without a predetermined sample size. Fifty psychologists/psychotherapists participated in the study. Their clinical experience was on average 4.8 years (*SD* = 5.0) and 96% had training in CBT. 42% reported having no previous knowledge about causal symptom networks.

#### Questionnaire: Case Conceptualization Criteria and Clinical Utility

Psychotherapists were presented with five randomly selected PECAN visualizations from Study 1. Each network was visualized using force-directed graphs. In these graphs, node size (the size of the circle) corresponds to problem severity, and edge width (the width of the arrows connecting circles) corresponds to perceived causality. For each network, weak relations were filtered out, with the cutoff set so that the total number of relations shown corresponded to the number of nodes in the network (this was done to decrease cluttering). While viewing the network, the respondent could adjust this filter cutoff, move nodes around to better understand the network, highlight feedback loops between items, and simulate hypothetical intervention effects by choosing a problem and exploring how intervention effects would spread in the network (indicated by decreasing node sizes). Note that respondents were not presented with numerical information about centrality, as this might easily be taken as an overly simplistic method of selecting treatment targets.

For each network, the psychotherapist rated on a Likert-type scale from 0 (*not at all*), over 3 (*correct*), to 6 (*extremely*) to which degree the network met the following criteria for a satisfactory client case conceptualization (inspired by Flitcroft et al., 2007):

- *Logical*: “The conceptualization makes sense, that is, it is understandable how the problems might cause each other.”
- *Identifiable targets*: “A small part of the network is particularly influential, causing most of the other problems.”
- *Explains maintenance*: “The influential part of the network includes a feedback loop, maintaining the network.”

In free-text questions, respondents were asked to select a part of the network that they would target in therapy, as well as what information was missing in the network to make it more clinically meaningful. Last, the respondents rated the proportion of information in the network (in %) compared with what typically is collected during a psychotherapy assessment phase.

After the presentation of the five randomly selected networks, the respondent was asked to select which of the following potential uses of PECAN were most likely helpful:

- “To prepare before meeting a client for the first time”
- “As a basis for discussion with a client”
- “As a basis for discussion with colleagues or with a supervisor”

Finally, respondents were asked to rate their general impression of the clinical utility of PECAN on a Likert-type scale from 0 (*not at all useful*), over 3 (*useful*), to 6 (*extremely useful*).

#### Data Analysis

All data are presented descriptively, without tests for significant differences. Due to some missing data, the 50 psychotherapists rated 247 networks.

### Results: Study 2

**Figure 2.**
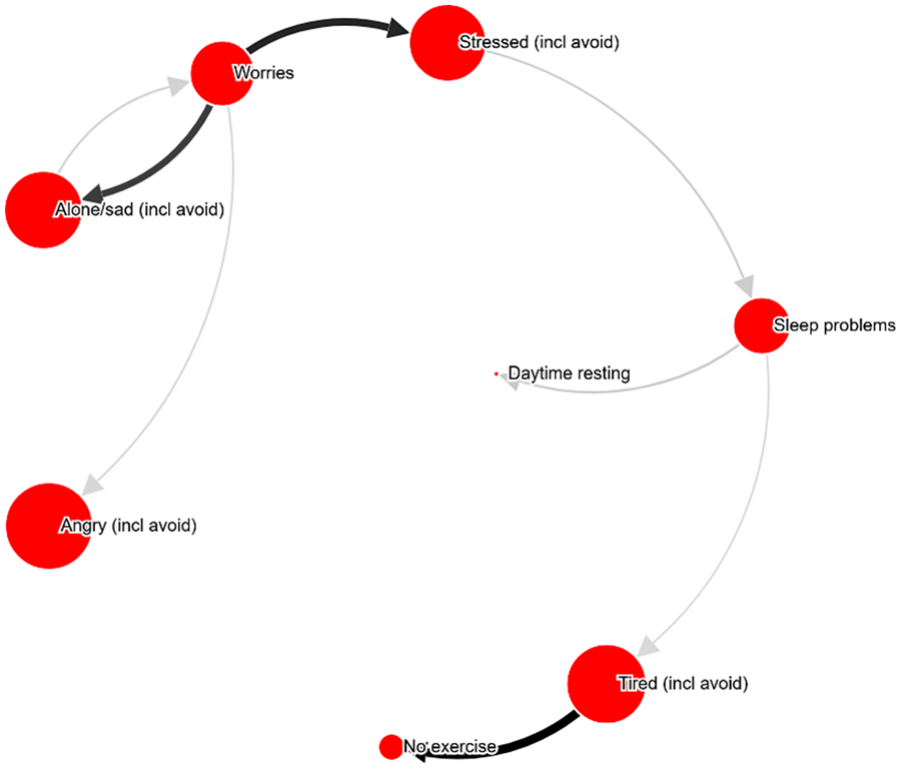
Example Network ID 160537 (50-59 years old; female), shown with relation cutoff at 7%. *Note*. Most central problem: Worries. An interactive version can be found at http://bit.ly/PECANfig2

Since networks were randomly selected, some networks were presented more than once. For illustrative purposes, the five networks that were presented four times or more are presented as Figures 2 through 6, and detailed information about them is provided in Table 3.

**Figure 3.**
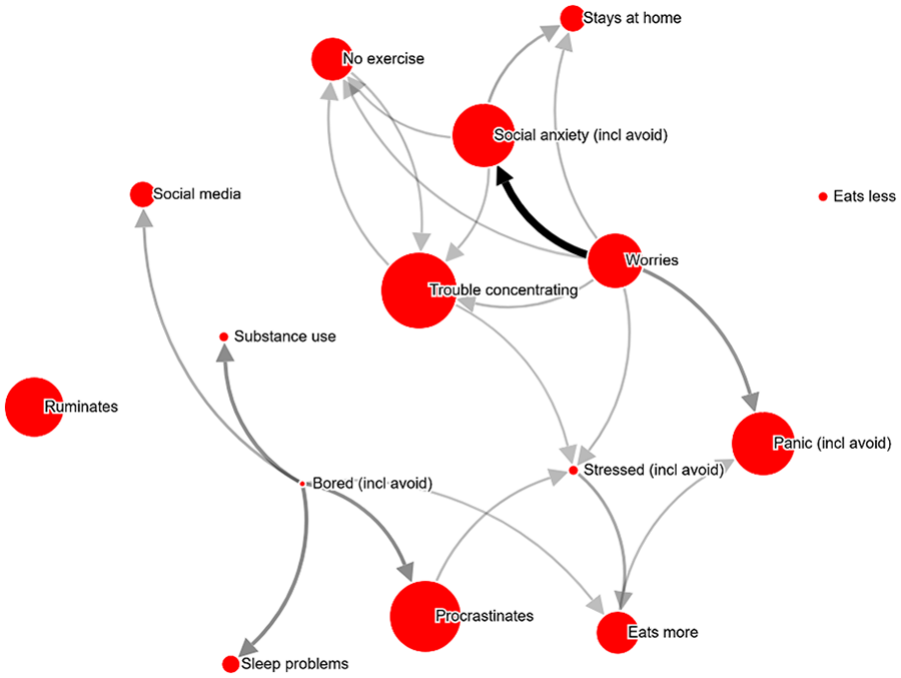
Example network ID 487943 (40-49 years old, male), shown with relation cutoff at 33%. *Note.* Most central problem: Worries. An interactive version can be found at http://bit.ly/PECANfig3

**Figure 4.**
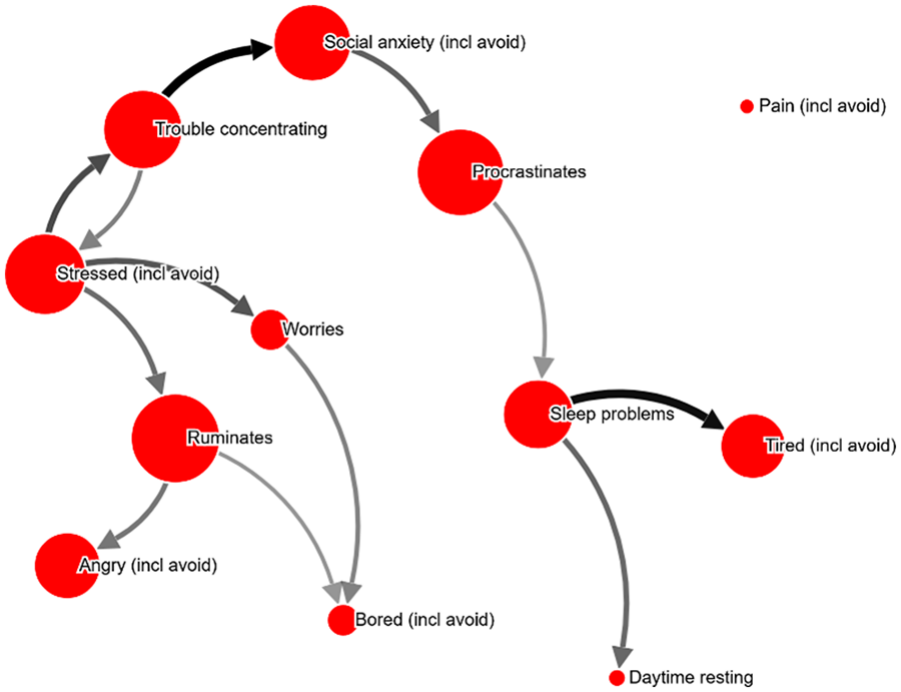
Example network ID 938110 (30-39 years old; female), shown with relation cutoff at 34%. *Note.* Most central problem: Trouble concentrating. An interactive version can be found at http://bit.ly/PECANfig4

**Figure 5.**
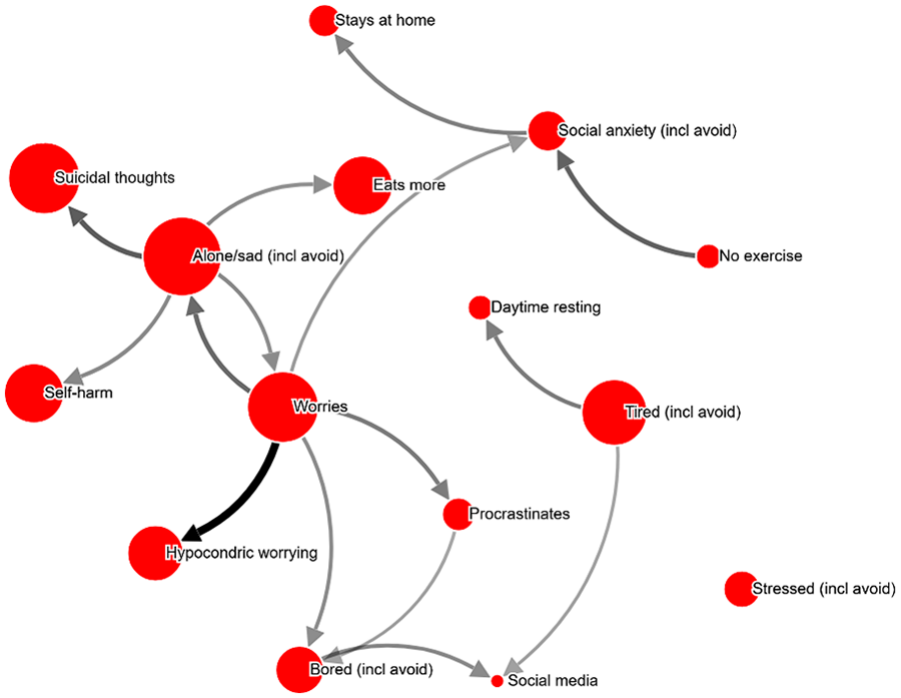
Example network ID 528589 (30-39 years old; female), shown with relation cutoff at 36%. *Note.* Most central problem: Worries. An interactive version can be found at http://bit.ly/PECANfig5

**Figure 6.**
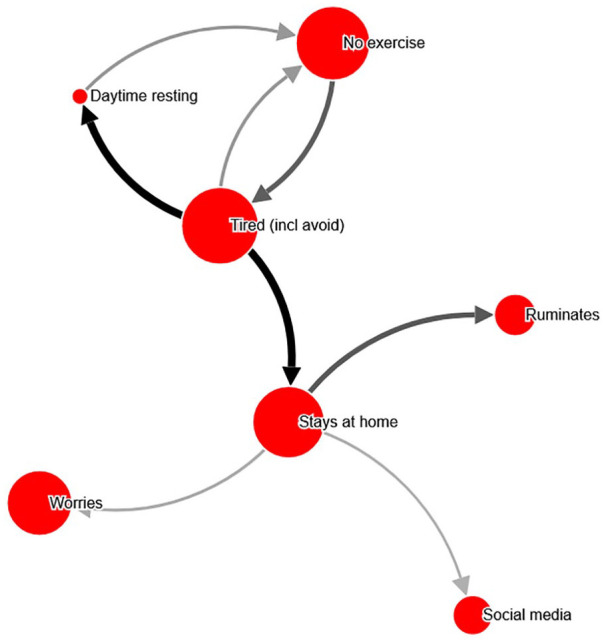
Example network ID 817339 (16-19 years old; female), shown with relation cutoff at 44%. *Note.* Most central problem: Tired. An interactive version can be found at http://bit.ly/PECANfig6

**Table 3.** Example PECAN visualizations (see Figures 2-6), selected based on being randomly presented more than 4 times to therapists.

Figure	PHQ-9 score	Test–retest	Therapist analyses (abbreviated; Question: “What part of the network would you target for an intervention, and how?”)
2	12	.62	*“Worries. Cognitive intervention.”* *“Worries. Postpone worry. Focus shift and attention training.”* *“Worry thoughts. Intervention: stimulus control. Behavioral activation with mindfulness.”* *“Worry. – Acceptance exercises, mindfulness.”* *“Worry. Hard to choose intervention without context.*
3	10	.85	*“Social anxiety and related worrying. Video feedback exercises for social anxiety.”* *“Worry, and investigate what situations trigger worry, and what the consequences are.”* *“Worrying-thoughts. Exposure.”* *“Worry. Intervention should be chosen based on frequency, situations, etc.* *“Relaxation”* *“Social anxiety. Exposure and cognitive restructuring.”*
4	10	.92	*“Stress, unclear what intervention would be suitable.”* *“Trouble concentrating. Challenge the meta-cognitions controlling attention.”* *“Worry-thoughts and social anxiety. Collect data on worry-content, then behavioral experiments.”* *“Possibly target trouble concentrating and rumination. An intervention to help control attention.”* *“Stress. How to intervene depends on what causes the stress.*
5	16	.77	*“Alone/sad: psychoeducation about emotions and emotion regulation strategies, behavioral activation with an emphasis on social relations. Worries: psychoeducation about emotions, postponing worry and problem-solving skills.”* *“Worries. Give the client a conceptualization of worry, and perhaps a GAD treatment.”* *“Again, worry seems to be what causes most of the other symptoms. An intervention should target worry-behaviors, stimulus control, acceptance strategies and problem solving.”* *“Worry and loneliness.”*
6	18	.89	*“Exercise”* *“Tired, but would have to know what’s causing the tiredness to choose an intervention.”* *“Passivity, i.e., the loop between ‘no exercise’ and ‘tired’. Behavioral activation.”* *“Tiredness—a sleep intervention or else try to get going with exercise.”*

#### Do PECAN Visualizations Meet Criteria for Being Useful Case Conceptualizations?

The proportion of presented networks that were scored as meeting the criteria for being a useful case conceptualization (a rating of 3 or above) were *Logical*: 59%, *Identifiable targets*: 66%, and *Explains maintenance*: 47%. On average, information contained in the presented networks were rated to cover 46.8% (*SD* = 24.4) of the information collected during a typical assessment phase in therapy.

#### What Is the Clinical Utility of the PECAN Method?

The mean utility-rating for the PECAN method was 4.2 (*SD* = 1.2), with 96% of psychotherapists rating PECAN as clinically useful (3 or higher). Eighty-nine percent selected “As a basis for discussion together with a client” as the most promising use of the method.

#### What Information Is Missing From PECAN?

The five most common types of information requests were:

- More patient-specific meaning of behavioral/emotional problems (e.g., “What topics do the patient worry about?”): 16%
- More information about contextual factors (e.g., “What social relationships do the patient have?”): 15%
- Causal relations that the psychotherapist would expect to find (e.g., “How does substance abuse affect the other ‘symptoms’?”: 14%
- External causes to problems (e.g., “Are there somatic causes for the pain?”: 12%
- Values, goals, or motivation (e.g., “What motivates this patient to change?”): 6%

In a post hoc analysis, we found that the median time for psychotherapists to review and respond to the questionnaire, divided by five networks, was 6.1 minutes per network. However, actual time per network was shorter, as this includes time to review study information and provide background information.

## Discussion

Previous literature has highlighted the need for individualized case conceptualizations to move beyond diagnosis-specific treatments to interventions that target processes relevant to the individual (Hayes & Hofmann, 2018). One possibility is to let patients rate how their behavioral and emotional problems are causally related (Frewen et al., 2012). Visualized idiosyncratic problem networks can then be created, which might be used as a first preparatory step in a personalized case conceptualization, and guiding treatment choices. In the present article, we expanded on previous methods for self-rated symptom networks and introduced the PECAN method, designed to generate clinically relevant case conceptualizations.

### Summary of Results

The PECAN method showed acceptable immediate test–retest reliability for item weighted outdegree centrality and responders completed the questionnaire (including retest items) quickly. Network structure varied across respondents, with every included behavioral/emotional problem being the most central problem for at least one respondent. However, one third reported that either worrying or ruminating was their most central problem (of note, neither worry nor rumination are diagnostic criteria of major depression).

Presenting the PECAN results to psychologists/psychotherapists, the networks were rated to contain on average 47% of the information typically collected during an assessment phase in therapy. Psychotherapists reported that in order for the method to become more clinically useful, the networks should contain contextual information (e.g., social situation), further specifications about behavioral/emotional problems (e.g., content of worrying), causes external to the network (e.g., somatic disorders or stressful environment), and client goals or values. Consequently, psychotherapists judged the PECAN to be most useful as a basis for a discussion with clients, which is in line with the tradition of case conceptualizations as a collaboration between therapist and client. Although these assessments by therapists tell us nothing about the actual validity of the idiosyncratic networks, it does indicate that therapists can see a clinical utility of such networks, and that they are considered a first step in a collaborative effort together with the client.

Compared with another self-report method, EMA, the PECAN is time-efficient, and may detect fine-grained causal relations that are hard to uncover using EMA. Compared with traditional case conceptualizations, the PECAN is less time-consuming for the psychotherapist, more structured, and empirically quantifiable. Results indicate that PECAN might be used as a first step in a more thorough and collaborative case conceptualization, in which the psychotherapist and client explore relations and feedback loops indicated by PECAN to guide treatment choices. Nevertheless, the PECAN method has not yet shown sufficient reliability, and more important, has not been evaluated for validity, to warrant its use as method to guide treatment choices.

### Limitations

First, depressed respondents were recruited through social media with no validation of diagnosis and are likely not representative of a clinical population. Second, only a third of those who initiated the PECAN questionnaire completed it, and these dropouts were likely systematic. For instance, previous studies have shown that about one third of depressed patients perceive their own behavior as the main cause of their disorder (Brown et al., 2007), and this belief is plausibly highly overrepresented in the present study. The low response rate might indicate that the questionnaire is too demanding and this may potentially limit its clinical use. Third, the causality ratings in the PECAN are likely to be systematically biased. Indeed, people tend to overestimate causality between behavioral phenomena (Gloster et al., 2017). This bias likely works in different directions for different behavior and emotions, so that respondents might overestimate how much insomnia causes concentration problems, and underestimate how much lack of exercise causes feeling tired. Fourth, other ways than weighted outdegree centrality to describe PECAN data might be more fruitful, perhaps identifying feedback loops that occur across several networks and use this to group networks. Fifth, it would be preferable to assess test–retest reliability over longer time periods. Although the reliability reported in this article might seem acceptable, one must bear in mind that this is reliability across only a few minutes—with delays across days or weeks this would obviously drop. Sixth, other analyses of reliability, such as interrater reliability between a client and family members, as suggested by Haynes et al. (2020) are also warranted. Finally, although psychologists/psychotherapists rated the clinical utility of PECAN as high, it is unknown whether this holds true in a real-life therapeutic situation. Related to this issue is whether allowing respondents to rate emotion-to-emotion causality does indeed increase clinical utility. Even if it does, which is debatable, the constraint of emotion-to-emotion causality used in the present study does limit to what extent the results represent the actual self-understanding of the respondents.

### Future Directions

The present study provides some suggestions on how the PECAN method could be improved. First, contextual factors need to be included, perhaps as nodes in the network or as options where respondents can provide free-text answers. Another interesting development of the method would be to include salutogenic behaviors/emotions as items. This could either be a mix of healthy and problematic items (and allowing causal relations to be either negative or positive, e.g., spending time with friends might decrease problematic behaviors such as rumination), or a version with only salutogenic items (i.e., precluding symptoms altogether). Furthermore, the list of emotional/behavioral problems included in the PECAN could be adapted to specific clinical populations, or could simply be expanded. As discussed above, the constraint of not allowing emotion-to-emotion causality could also be removed. Also, the method could be used to create average networks for groups of patients, in which case low reliability on the individual level can be countered by more respondents (thus returning to the nomothetic approach).

Finally, given the somewhat low reliability (given that the retest assessment was immediate and not delayed), it might be fruitful not to rely on a single assessment of perceived causality between problems, but rather combining this method with a more ecological methodology. For instance, a respondent could be asked at one random time point every day across a few weeks which problem he or she is experiencing at that moment, and what other problems are causing those problems. Then, an average across many such ecological assessments of perceived causality can be created and visualized.

Regarding the perception of the PECAN method by psychologists/psychotherapists, PECAN was reported to be most useful as a basis for discussion with the client. Thus, an interesting way forward is to optimize the method for this purpose, perhaps by adding another step in the methodology in which the psychotherapist and client modify the network in collaboration, adding, removing, and/or adjusting behavioral/emotional problems and specific causal relations. This particular use of the PECAN could be evaluated by assessing whether this increases client motivation or client–therapist agreement on the most promising interventions, or as a preparatory step before data collection using EMA.

In an interesting study by Rubel et al. (2018), expert psychotherapists rated the degree to which different interventions would impact specific nodes in a symptom network. By multiplying the centrality of each problem with each intervention’s specific effects, each intervention was given a priority ranking. This could be combined with PECAN to help psychotherapists select between interventions. However, with this follows the risk of an overreliance on node centrality, which has been criticized (Bringmann et al., 2019). Instead, perceived networks could be used as a first step to simulating how an idiosyncratic network might work, and how interventions targeting one or many nodes simultaneously would affect the full network (Henry et al., 2020).

The million-euro question is of course—validity. One method to test the validity of a case conceptualization is to test the intervention suggested by such a conceptualization, then assess whether the expected generalized treatment effects follows. This would indicate at least clinical utility. Another option, suggested by Mumma et al. (2018) is to validate the PECAN against EMA methods. Again, intervention effects should not be expected to spread in a linear fashion across the network, and simulation of networks is likely needed to predict what treatment effects can be expected (Burger et al., 2020; Henry et al., 2020).

In sum, the PECAN methodology shows promise as a time-efficient first step when designing a client-specific case conceptualization. Future research should explore ways to improve the method further and assess the reliability, validity, and utility of the method in clinical settings.

## Supplemental Material

sj-csv-1-asm-10.1177_10731911211039281 – Supplemental material for Perceived Causal Problem Networks: Reliability, Central Problems, and Clinical Utility for Depression Click here for additional data file. Supplemental material, sj-csv-1-asm-10.1177_10731911211039281 for Perceived Causal Problem Networks: Reliability, Central Problems, and Clinical Utility for Depression by Lars Klintwall, Martin Bellander and Matti Cervin in Assessment

sj-csv-2-asm-10.1177_10731911211039281 – Supplemental material for Perceived Causal Problem Networks: Reliability, Central Problems, and Clinical Utility for Depression Click here for additional data file. Supplemental material, sj-csv-2-asm-10.1177_10731911211039281 for Perceived Causal Problem Networks: Reliability, Central Problems, and Clinical Utility for Depression by Lars Klintwall, Martin Bellander and Matti Cervin in Assessment

sj-csv-3-asm-10.1177_10731911211039281 – Supplemental material for Perceived Causal Problem Networks: Reliability, Central Problems, and Clinical Utility for Depression Click here for additional data file. Supplemental material, sj-csv-3-asm-10.1177_10731911211039281 for Perceived Causal Problem Networks: Reliability, Central Problems, and Clinical Utility for Depression by Lars Klintwall, Martin Bellander and Matti Cervin in Assessment

sj-csv-4-asm-10.1177_10731911211039281 – Supplemental material for Perceived Causal Problem Networks: Reliability, Central Problems, and Clinical Utility for Depression Click here for additional data file. Supplemental material, sj-csv-4-asm-10.1177_10731911211039281 for Perceived Causal Problem Networks: Reliability, Central Problems, and Clinical Utility for Depression by Lars Klintwall, Martin Bellander and Matti Cervin in Assessment This article is distributed under the terms of the Creative
Commons Attribution 4.0 License ( https://creativecommons.org/licenses/by/4.0/) which
permits any use, reproduction and distribution of the work without
further permission provided the original work is attributed as specified
on the SAGE and Open Access pages ( https://us.sagepub.com/en-us/nam/open-access-at-sage).

sj-docx-1-asm-10.1177_10731911211039281 – Supplemental material for Perceived Causal Problem Networks: Reliability, Central Problems, and Clinical Utility for Depression Click here for additional data file. Supplemental material, sj-docx-1-asm-10.1177_10731911211039281 for Perceived Causal Problem Networks: Reliability, Central Problems, and Clinical Utility for Depression by Lars Klintwall, Martin Bellander and Matti Cervin in Assessment This article is distributed under the terms of the Creative
Commons Attribution 4.0 License ( https://creativecommons.org/licenses/by/4.0/) which
permits any use, reproduction and distribution of the work without
further permission provided the original work is attributed as specified
on the SAGE and Open Access pages ( https://us.sagepub.com/en-us/nam/open-access-at-sage).

sj-pdf-1-asm-10.1177_10731911211039281 – Supplemental material for Perceived Causal Problem Networks: Reliability, Central Problems, and Clinical Utility for Depression Click here for additional data file. Supplemental material, sj-pdf-1-asm-10.1177_10731911211039281 for Perceived Causal Problem Networks: Reliability, Central Problems, and Clinical Utility for Depression by Lars Klintwall, Martin Bellander and Matti Cervin in Assessment

sj-pdf-2-asm-10.1177_10731911211039281 – Supplemental material for Perceived Causal Problem Networks: Reliability, Central Problems, and Clinical Utility for Depression Click here for additional data file. Supplemental material, sj-pdf-2-asm-10.1177_10731911211039281 for Perceived Causal Problem Networks: Reliability, Central Problems, and Clinical Utility for Depression by Lars Klintwall, Martin Bellander and Matti Cervin in Assessment

sj-pdf-3-asm-10.1177_10731911211039281 – Supplemental material for Perceived Causal Problem Networks: Reliability, Central Problems, and Clinical Utility for Depression Click here for additional data file. Supplemental material, sj-pdf-3-asm-10.1177_10731911211039281 for Perceived Causal Problem Networks: Reliability, Central Problems, and Clinical Utility for Depression by Lars Klintwall, Martin Bellander and Matti Cervin in Assessment

sj-rar-1-asm-10.1177_10731911211039281 – Supplemental material for Perceived Causal Problem Networks: Reliability, Central Problems, and Clinical Utility for Depression Click here for additional data file. Supplemental material, sj-rar-1-asm-10.1177_10731911211039281 for Perceived Causal Problem Networks: Reliability, Central Problems, and Clinical Utility for Depression by Lars Klintwall, Martin Bellander and Matti Cervin in Assessment This article is distributed under the terms of the Creative
Commons Attribution 4.0 License ( https://creativecommons.org/licenses/by/4.0/) which
permits any use, reproduction and distribution of the work without
further permission provided the original work is attributed as specified
on the SAGE and Open Access pages ( https://us.sagepub.com/en-us/nam/open-access-at-sage).
