# Supplementary material for: Perceived Causal Problem Networks: Reliability, Central Problems, and Clinical Utility for Depression
Source: Assessment. 2021 Sep 1;30(1):73–83. doi: 10.1177/10731911211039281 (PMC9684655; doi:10.1177/10731911211039281)
Supplement: sj-docx-1-asm-10.1177_10731911211039281 – Supplemental material for Perceived Causal Problem Networks: Reliability, Central Problems, and Clinical Utility for Depression [file sj-docx-1-asm-10.1177_10731911211039281.docx]

Swedish-english translation

Äter mindre – eats less

Tränar inte – no exercise

Somnar sent - insomnia

Vilar – daytime resting

Bråkar - conflicts

Sjukdomstankar – hypocondric worry

Fokuserar inte – unfocused

Sociala medier – social media use

Stannar hemma – stays at home

Skjuter upp - procrastinates

Alkohol/droger – alcohol / drugs

Självskadar – self-harm

Självmordstankar – suicidal thoughts

Äter mer - overeats

Tvångar – compulsive behaviors

Ältar - rumination

Orostankar - worry

Flashbacks - flashbacks

Panikångest - panic

Smärta – somatic pain

Social ångest -social anxiety

Ensam/ledsen – alone/sad

Trött - tired

Stressad - stress

Uttråkad - bored

Arg - angry
