# Supplementary material for: Perceived Causal Problem Networks: Reliability, Central Problems, and Clinical Utility for Depression
Source: Assessment. 2021 Sep 1;30(1):73–83. doi: 10.1177/10731911211039281 (PMC9684655; doi:10.1177/10731911211039281)
Supplement: sj-pdf-1-asm-10.1177_10731911211039281 – Supplemental material for Perceived Causal Problem Networks: Reliability, Central Problems, and Clinical Utility for Depression [file sj-pdf-1-asm-10.1177_10731911211039281.pdf]

Appendix 1. Behavior/emotional problems included in the PECAN, with different phrasings in the questionnaire. All items were in Swedish.

**Behavior/emotional problem (short-hand)**	**When presented as problem to be explained: "In the past week, why did you - "**	**When presented as possible cause to other problems (both for selecting and % ratings)**
Eats less	- skip meals?	I had skipped meals
No exercise	- opt out on physical exercise?	I don't exercise
Sleep problems	- fall asleep late or didn't sleep well	I got too little sleep the previous night
Daytime resting	- rest or sleep during daytime?	I had slept or rested during daytime
Conflicts	- get into fights / arguments with family or friends?	I had been in a fight with family or friends
Hypocondric worries	- scan your body for - / google symptoms?	I was worried about being sick
Trouble concentrating	- have trouble focusing or make decisions?	I could not focus my thoughts or come to a decision
Social media	- get stuck on social media?	I got stuck on social media
Stays at home	- stay home from work, school or seeing people?	I stayed home from work, school or seeing people
Procrastinates	- postpone or avoid chores or assignments?	I had postponed or avoided chores or assignments
Substance use	- use alcohol or drugs?	I had used alcohol / drugs
Self-harm	- hurt your own body?	I had hurt my own body
Suicidal thoughts	- consider, plan, or attempt to kill yourself?	I considered, planned or tried to commit suicide
Eats more	- eat more than you should?	I had eaten too much
Compulsions	- do compulsive behaviors?	I got stuck in, or wanted to avoid, compulsive behaviors
Ruminates	- get stuck in rumination (past events, self-blame)	I got stuck in, or wanted to avoid, ruminative thoughts
Worries	- get stuck in worrying (economy, friends, disaster)	I got stuck in, or wanted to avoid, worrying thoughts
Flashbacks (incl avoid)	- experience intrusive memories from traumas?	I had, or wanted to avoid, traumatic flashbacks
Panic (incl avoid)	- have panic-like anxiety?	I had, or wanted to avoid, panic like anxiety
Pain (incl avoid)	- felt pain (headache, stomachache)?	I had, or wanted to avoid, pain (headache/stomachache)
Social anxiety (incl avoid)	- experience social anxiety?	I had, or wanted to avoid, social anxiety
Alone/sad (incl avoid)	- feel painfully alone or sad?	I felt, or wanted to avoid feeling, lonely or sad
Tired (incl avoid)	- feel extremely tired?	I felt, or wanted to avoid feeling, tired
Stressed (incl avoid)	- feel stressed out or hyper	I felt, or wanted to avoid feeling, stressed out
Bored (incl avoid)	- feel bored or inability to experience joy?	I felt, or wanted to avoid feeling, bored or lack of joy
Angry (incl avoid)	- feel angry / irritable?	I felt, or wanted to avoid feeling, angry
