# Supplementary material for: Perceived Causal Problem Networks: Reliability, Central Problems, and Clinical Utility for Depression
Source: Assessment. 2021 Sep 1;30(1):73–83. doi: 10.1177/10731911211039281 (PMC9684655; doi:10.1177/10731911211039281)
Supplement: sj-pdf-2-asm-10.1177_10731911211039281 – Supplemental material for Perceived Causal Problem Networks: Reliability, Central Problems, and Clinical Utility for Depression [file sj-pdf-2-asm-10.1177_10731911211039281.pdf]

AVERAGE	Eats less	No exercise	Insomnia	Resting	Conflicts	Hypocondria	Unfocused	social media	Stay shome	Procrast	Substances	Selfharm	Suicidal	Eats more	Compulsions	Ruminate	Worry	Flashback/avoid	Panic/avoid	Pain/avoid	Social anxiety/avoid	Alone/sad/avoid	Tired/avoid	Stressed/avoid	Bored/avoid	Angry/avoid
Eats less		10	3	4	0	9	8	0	0	5	7	7	0	7	1	0	0	5	4	15	9	13	5	3	6	19
No exercise	2	2		11	4	10	2	4	4	6	1	8	6	4	4	3	8	11	2	5	0	0	0	1	3	7
Insomnia	0	0	1	4		5	8	9	4	9	6	2	0	17	0	1	7	11	4	9	7	0	0	13	6	3
Resting	1	3	8	8	37	4		5	9	4	3	3	1	2	3	7	8	0	8	1	7	0	0	12	5	1
Conflicts	0	0	0	0	2	8	2	8		0	0	1	0	1	1	0	0	4	2	18	8	0	0	0	0	0
Hypocondria	0	0	0	9	2	9	1	4	0	0		7	3	0	0	10	0	0	0	0	0	0	0	0	0	3
Unfocused	4	4	4	2	14	8	0	9	0	7	3	1		4	7	2	4	4	3	2	1	0	0	3	2	3
social media	0	0	2	7	1	4	2	0	0	9	0	0	14	6		9	4	12	9	6	0	0	0	6	6	0
Stay shome	4	3	0	0	7	3	1	7	5	7	10	0	8	1	0	0		0	5	0	0	0	0	3	8	1

Procrast	1	7	0	4	6	1	8	8	6	1	3	3	30	6	10	8	2	8		4	1	0	0	6	4	0
Substances	0	0	1	2	1	3	0	0	8	5	1	9	2	9	0	0	0	0	0	7		4	7	26	4	0
Selfharm	0	0	0	0	0	0	0	0	0	0	0	0	3	4	11	3	0	0	3	7	14	3		23	3	6
Suicidal	1	5	3	4	0	0	0	0	22	0	0	0	2	0	1	7	0	0	1	1	13	3	0	0		3
Eatsmore	18	8	5	3	2	0	0	0	3	1	6	4	6	3	0	5	3	3	0	7	0	0	6	0	7	7
Compulsions	0	0	0	0	0	0	0	0	0	0		5	0	0	0	5	0	0	0	0	0	0	0	0	0	
Ruminate	0	0	3	3	4	1	0	4	14	4	11	3	8	3	3	9	6	4	5	8	4	5	3	9	6	9
Worry	1	4	3	8	5	7	0	7	5	6	19	1	9	4	0	9	4	1	4	6	2	6	9	6	8	9
Flashback/avoid	0	5	1	1	6	3	0	0	17	9	0	0	2	1	0	0	6	6	0	7	0	0	11	0	16	4
Panic/avoid	1	9	2	7	7	7	2	4	15	6	28	7	8	6	0	0	6	8	10	1	3	6	13	8	11	7
Pain/avoid	20	4	15	7	21	0	2	0	4	9	14	3	4	1	1	2	3	6	3	7	5	4	14	3	7	2

Social anxiety/avoid	1	5	1	7	1	8	0	0	3	6	15	8	11	0	0	3	5	5	1	6	0	0	0	0	9	6
Alone/sad/avoid	1	7	3	4	8	1	0	0	14	7	9	9	6	4	3	8	12	8	1	8	6	9	9	7	15	7
Tired/avoid	8	7	15	0	40	6	3	8	9	6	8	0	5	7	2	6	4	3	3	8	11	6	0	0	7	5
Stressed/avoid	4	3	8	3	8	7	1	8	11	1	9	2	18	7	3	7	3	6	13	3	0	0	9	5	4	7
Bored/avoid	0	0	7	1	12	0	2	6	8	6	8	4	11	9	7	8	7	7	10	7	0	0	0	0	7	0
Angry/avoid	5	1	3	4	14	5	0	0	15	6	5	0	12	7	6	1	5	3	3	4	1	6	2	4	0	8
