# Supplementary material for: Perceived Causal Problem Networks: Reliability, Central Problems, and Clinical Utility for Depression
Source: Assessment. 2021 Sep 1;30(1):73–83. doi: 10.1177/10731911211039281 (PMC9684655; doi:10.1177/10731911211039281)
Supplement: sj-pdf-3-asm-10.1177_10731911211039281 – Supplemental material for Perceived Causal Problem Networks: Reliability, Central Problems, and Clinical Utility for Depression [file sj-pdf-3-asm-10.1177_10731911211039281.pdf]

AVERAGE	Eats less	No exercise	Insomnia	Resting	Conflicts	Hypocondria	Unfocused	social media	Stayshome	Procrast	Substances	Selfharm	Suicidal	Eats more	Compulsions	Ruminate	Worry	Flashback/avoid	Panic/avoid	Pain/avoid	Social anxiety/avoid	Alone/sad/
Eats less		10,3	4,0	9,8	0,0	5,7	7,0	7,1	0,0	5,4	15,9	13,5	3,6	19,2	13,1	5,6	7,5	6,4	11,3	6,8	4,0	7,4
No exercise	2,2		11,4	10,2	4,4	6,1	8,6	4,4	3,8	11,2	5,0	0,0	1,3	7,5	0,0	7,0	4,9	6,9	4,7	13,2	6,9	6,4
Insomnia	0,0	1,4		5,8	9,4	9,6	2,0	17,0	1,7	11,4	9,7	0,0	13,6	3,3	14,3	17,1	18,3	22,0	13,7	5,0	5,5	15,8
Resting	1,3	8,8	37,4		5,9	4,3	3,1	2,3	7,8	0,8	1,7	0,0	12,5	1,0	12,5	4,4	5,5	1,7	9,9	9,5	3,2	5,3
Conflicts	0,0	0,0	2,8	2,8		0,0	1,0	1,1	0,0	4,2	18,8	0,0	0,0	0,0	0,0	8,6	14,8	0,0	7,8	0,0	1,4	12,4
Hypocondria	0,0	0,9	2,9	1,4	0,0		7,3	0,0	10,0	0,0	0,0	0,0	0,0	3,2		12,2	19,7	0,0	17,0	42,7	0,0	0,0
Unfocused	4,4	4,2	14,8	0,9	0,7	3,1		4,7	2,4	4,3	2,1	0,0	3,2	3,1	28,2	15,4	16,1	9,8	10,5	7,8	10,8	8,2
social media	0,0	2,7	1,4	2,0	0,9	0,0	14,6		9,4	12,9	6,0	0,0	6,6	0,3	8,7	16,3	18,4	3,9	3,0	2,8	7,0	13,5
Stayshome	4,3	0,0	7,3	1,7	5,7	10,0	8,1	0,0		0,5	0,0	0,0	3,8	1,1	22,3	4,7	5,4	26,6	9,5	15,3	20,5	23,0
Procrast	1,7	0,4	6,1	8,8	6,1	3,3	30,6	10,8	2,8		4,1	0,0	6,4	0,0	13,8	7,8	14,3	0,8	9,6	7,0	3,9	7,3
Substances	0,0	1,2	1,3	0,0	8,5	1,9	2,9	0,0	0,0	0,7		4,7	26,4	0,7	25,0	13,3	18,1	27,0	24,0	4,6	11,3	21,6
Selfharm	0,0	0,0	0,0	0,0	0,0	0,0	3,4	11,3	0,0	3,7	14,3		23,3	6,6	0,0	43,5	13,0	19,3	22,0	7,1	0,0	18,6
Suicidal	1,5	3,4	0,0	0,0	22,0	0,0	2,0	1,7	0,0	1,1	13,3	0,0		3,6	34,8	30,0	26,6	23,1	10,3	4,8	3,1	25,8
Eats more	18,8	5,3	2,0	0,0	3,1	6,4	6,3	0,5	3,3	0,7	0,0	6,0	7,7			12,7	10,1	3,6	2,9	3,5	5,9	28,0
Compulsions	0,0	0,0	0,0	0,0	0,0		5,0	0,0	5,0	0,0	0,0	0,0	0,0			22,3	29,4	0,0	0,0	0,0	2,3	5,0
Ruminate	0,0	3,3	4,1	0,4	14,4	11,3	8,3	3,9	6,4	5,8	4,5	3,9	6,9	3,2	0,0		19,7	24,6	6,8	5,7	12,8	25,5
Worry	1,4	3,8	5,7	0,7	5,6	19,1	9,4	0,9	4,1	4,6	2,6	9,6	8,9	5,1	12,7	17,7		17,3	11,6	3,8	9,5	16,1
Flashback/avoid	0,5	1,1	6,3	0,0	17,9	0,0	2,1	0,0	6,6	0,7	0,0	11,0	16,4	0,0	0,0	26,3	25,1					
Panic/avoid	1,9	2,7	7,7	2,4	15,6	28,7	8,6	0,0	6,8	10,1	3,6	13,8	11,7	5,3	0,0	21,7	34,3					
Pain/avoid	20,4	15,7	21,0	2,0	4,9	14,3	4,1	1,2	3,6	3,7	5,4	14,3	7,2	3,6	0,0	13,4	21,9					
Social anxiety/avoid	1,5	1,7	1,8	0,0	3,6	15,8	11,0	0,3	5,5	1,6	0,0	0,0	9,6	7,0	0,0	27,1	19,7					
Alone/sad/avoid	1,7	3,4	8,1	0,0	14,7	9,9	6,4	3,8	12,8	1,8	6,9	9,7	15,7	6,2	4,6	32,0	21,2					
Tired/avoid	8,7	15,0	40,6	3,8	9,6	8,0	5,7	2,6	4,3	3,8	11,6	0,0	7,5	2,8	11,1	13,8	14,7					
Stressed/avoid	4,3	8,3	8,7	1,8	11,1	9,2	18,7	3,7	3,6	13,3	0,0	9,5	4,7	2,7	22,1	18,9	26,4					
Bored/avoid	0,0	7,1	12,0	2,6	8,6	8,4	11,9	7,8	7,7	10,7	0,0	0,0	7,0	1,1	14,4	21,0	19,0					
Angry/avoid	5,1	3,4	14,5	0,0	15,6	5,0	12,7	6,1	5,3	3,4	1,6	2,4	0,8	7,1	0,0	20,8	20,1					
